# The Contribution of Anterior Segment Abnormalities to Changes in Intraocular Pressure in the DBA/2J Mouse Model of Glaucoma: DBA/2J-*Gpnmb*^+^/SjJ Mice as Critical Controls

**DOI:** 10.3389/fnins.2021.801184

**Published:** 2022-02-03

**Authors:** Landon J. Rohowetz, Marc E. Mardelli, R. Scott Duncan, Sean M. Riordan, Peter Koulen

**Affiliations:** ^1^Department of Ophthalmology, Vision Research Center, School of Medicine, University of Missouri – Kansas City, Kansas City, MO, United States; ^2^Department of Biomedical Sciences, School of Medicine, University of Missouri—Kansas City, Kansas City, MO, United States

**Keywords:** glaucoma, retina, iris pigment dispersion, intraocular pressure, iris stromal atrophy, cornea, corneal calcification, anterior chamber

## Abstract

The contributions of anterior segment abnormalities to the development of ocular hypertension was determined in the DBA/2J mouse model of glaucoma. Intraocular pressure (IOP) was measured non-invasively. Iris pigment dispersion (IPD) and corneal calcification were measured weekly starting at 20 weeks of age in DBA/2J and DBA/2J-*Gpnmb*^+^/SjJ mice. Thickness, surface area, auto-fluorescence intensity, and perimeter length of calcified regions were measured in postmortem corneas using confocal microscopy. DBA/2J mice developed elevated IOP between 9 and 12 months of age, but DBA/2J-*Gpnmb*^+^/SjJ mice did not. Corneal calcification was found at all ages observed and at similar frequencies in both strains with 83.3% of DBA/2J eyes and 60.0% of DBA/2J-*Gpnmb*^+^/SjJ eyes affected at 12 months (*P* = 0.11). Calcification increased with age in both DBA/2J (*P* = 0.049) and DBA/2J-*Gpnmb*^+^/SjJ mice (*P* = 0.04) when assessed qualitatively and based on mixed-effects analysis. No differences in the four objective measures of calcification were observed between strains or ages. At 12 months of age, DBA/2J mice with corneal calcification had greater mean IOP than DBA/2J mice without corneal calcification. IOP was not correlated with the qualitatively assessed measures of calcification. For the subset of eyes with ocular hypertension, which were only found in DBA/2J mice, IOP was negatively correlated with the qualitative degree of calcification, but was not correlated with the four quantitative measures of calcification. Differences in IOP were not observed between DBA/2J-*Gpnmb*^+^/SjJ mice with and without calcification at any age. IPD increased with age and demonstrated a moderate correlation with IOP in DBA/2J mice, but was not observed in DBA/2J-*Gpnmb*^+^/SjJ mice. In the DBA/2J mouse model of glaucoma, increased IPD is positively correlated with an increase in IOP and corneal calcification is present in the majority of eyes at and after age 9 months. However, while IPD causes ocular hypertension, corneal calcification does not appear to contribute to the elevation of IOP, as the control strain DBA/2J-*Gpnmb*^+^/SjJ exhibits corneal calcification similar to DBA/2J mice, but does not develop ocular hypertension. Corneal calcification, therefore, does not appear to be a contributing factor to the development of elevated IOP in DBA/2J mice.

## Introduction

Glaucoma is characterized by progressive dysfunction and degeneration of the optic nerve and is the leading cause of irreversible blindness worldwide, affecting 64.3 million individuals in 2013 (Tham et al., 2014). Experimental models of glaucoma are important to understanding the disease and identifying potential therapeutic strategies. The DBA/2J inbred mouse is one of the most widely used models of ocular hypertension-induced glaucoma and was first described as a model for glaucoma in 1995 (Sheldon et al., 1995; Inman et al., 2006; Schlamp et al., 2006; Burroughs et al., 2011; Bosco et al., 2018; Mathieu et al., 2018; Buchanan et al., 2019; Jassim et al., 2019). DBA/2J mice develop severe iris pigment dispersion (IPD), which causes elevated intraocular pressure (IOP) as a result of occluded aqueous humor drainage pathways (John et al., 1998; Libby et al., 2005; Scholz et al., 2008). IPD occurs in DBA/2J mice due to a mutation in the glycoprotein (transmembrane) nmb gene (*Gpnmb^R150X^)*. A separate iris abnormality, iris stromal atrophy (ISA), occurs as a result of a mutation in the tyrosinase related protein 1 gene (*Tyrp1^b^*) (Chang et al., 1999; Anderson et al., 2002; Libby et al., 2005; Howell et al., 2007). Homozygosity for both of these genes accounts for the severe iris abnormalities including ISA and IPD seen in DBA/2J mice (Anderson et al., 2002).

In addition to these factors rendering DBA/2J mice a model of ocular hypertension-induced glaucoma, DBA/2J mice are also predisposed to a variety of systemic disorders including seizures, high-frequency hearing loss, thoracic cavity malformation and dystrophic calcification (van den Broek and Beynen, 1998; Shin et al., 2010; Jackson et al., 2015; Turner et al., 2017). Evidence of calcification has been reported in several tissues and organs throughout the body, including the heart, skeletal muscle, tongue, kidney, testes and diaphragm (van den Broek and Beynen, 1998). The cornea is also a common site for calcification in DBA/2J mice that increases in incidence and severity with age (John et al., 1998; Inman et al., 2006; Chou et al., 2011; Rutsch et al., 2011; Bricker-Anthony and Rex, 2015). This is particularly important for the use of DBA/2J mice as a glaucoma model, as calcification may impact non-invasive IOP measurement due to its effects on corneal thickness and elasticity (Brandt, 2001; Turner et al., 2017). Indeed, when compared with invasive IOP measurement, non-invasive measurement of IOP has been shown to be unreliable and often falsely elevated in DBA/2J mice (Turner et al., 2017). Furthermore, in DBA/2J mice, increased corneal thickness has been associated with elevated IOP measured non-invasively (Inman et al., 2006; Chou et al., 2011). However, despite these shortcomings non-invasive rebound tonometry continues to be used in mouse studies. Non-invasive measurement is a more humane method of measurement, it does not require anesthesia, and it is essential to longitudinal and histological studies where damage to the eye due to measurement is unacceptable.

IOP- and age-dependent decreases in visual acuity are characteristic components of glaucoma disease progression in DBA/2J mice (Wong and Brown, 2007; Burroughs et al., 2011; Grillo et al., 2013; Kaja et al., 2014; Grillo and Koulen, 2015; Montgomery et al., 2016; Yang et al., 2018). At the same time, adequate fundus and retinal imaging can be difficult to obtain due to anterior chamber abnormalities, including corneal calcification, in DBA/2J mice (Turner et al., 2017). In addition, other forms of corneal pathology have been identified in DBA/2J mice, including ulcers, erosions, neovascularization and basement membrane mineralization (John et al., 1998; Inman et al., 2006). Given the extent and severity of corneal and anterior chamber abnormalities in DBA/2J mice, the goal of the present study was to determine the relative contributions of such abnormalities to the development of ocular hypertension in the DBA/2J mouse model of glaucoma. A recently described control strain for DBA/2J mice, DBA/2J-*Gpnmb*^+^/SjJ, is characterized by a functional *Gpnmb* allele. Although these mice develop very mild ISA due to the homozygous mutation in *Tyrp1^b^*, they do not develop IPD, elevated IOP, or glaucoma and therefore have been suggested as a more closely matched control for DBA/2J mice (Howell et al., 2007). Therefore, we employed a comparison of the DBA/2J and DBA/2J-*Gpnmb*^+^/SjJ mouse strains to determine the relative roles of age-related changes in IOP and corneal pathology, specifically corneal calcification in the DBA/2J mouse model of glaucoma.

## Materials and Methods

### Animals

DBA/2J (*n* = 30) and DBA/2J-*Gpnmb*^+^/SjJ (*n* = 30) mice were purchased from Jackson Laboratories (Bar Harbor, ME). Mice were socially housed with *ad libitum* access to food and water and were kept under a 12-h dark/light cycle. 10 mice from each strain were euthanized at 6, 9, or 12 months of age. One DBA/2J animal from the 12-month group was lost to sampling and follow-up. All animal husbandry and experimental procedures had been approved by the Institutional Animal Care and Use Committee and were conducted in compliance with the Public Health Service Policy on Humane Care and Use of Laboratory Animals and in accordance with the ARVO Animal Statement and institutional guidelines.

### Measurement of Intraocular Pressure

As validated previously (Pease et al., 2011), IOP was measured weekly starting at 9 weeks of age until the end of the study with rebound tonometry (Icare TONOLAB, Colonial Medical Supply Co., Inc., Franconia, NH) not requiring anesthesia. Three measurements, each consisting of the average of six repeated measurements performed by the tonometry system, were obtained in each eye and the average was used for analysis.

### Qualitative Assessment of Structural Changes of the Cornea and Iris

**FIGURE 1:**
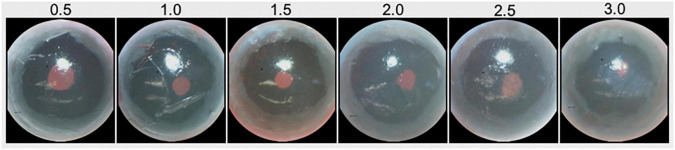
Qualitative measures of corneal calcification in DBA/2J and DBA/2J-*Gpnmb*^+^/SjJ mice. Brightness and contrast have been adjusted to increase visibility of calcifications.

A qualitative assessment of corneal calcification and IPD was conducted weekly beginning at 20 weeks of age by the same investigator, who was blinded to the strain identity or age of mice. Imaging of the anterior segment and for evaluation of corneal calcification and IPD was performed non-invasively and without the need for anesthesia. The investigator used the grading system illustrated in Table 1 and Figure 1 for corneal calcification and as described previously for IPD (Swaminathan et al., 2013). As the pupillary light reflex in some animals becomes diminished over time, such qualitative assessment, while both biologically and clinically relevant due to their non-invasiveness and ease-of-use in longitudinal studies, were complemented with quantitative measures as follows.

**TABLE 1:** Qualitative corneal calcification grading system.

Severity of corneal calcification	Observation
0.5	Calcification visible. Multiple observations needed to confirm presence of calcification.
1.0	Calcification approaches edge of constricted pupil but did not overlap
1.5	Calcification bordered constricted pupil
2.0	Calcification ended within constricted pupil
2.5	Calcification crossed half of constricted pupil
3.0	Calcification crossed at least three–fourths of constricted pupil

*Measurements were obtained weekly in all mice beginning at 20 weeks of age. Large calcifications across the top or bottom of pupil were down-graded by 0.5 points and severely opaque or vascularized small calcifications were upgraded by 0.5 points.*

### Quantitative Assessment of Structural Changes of Cornea Calcification

**FIGURE 2:**
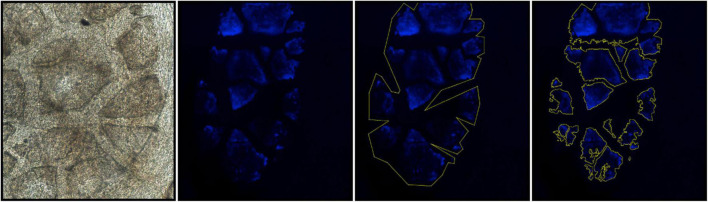
Quantitative measures of corneal calcification in DBA/2J and DBA/2J-*Gpnmb*^+^/SjJ mice. Image-J/FIJI was used to generate maximum intensity projections of corneal autofluorescence. Calcified regions of interest (ROIs) were isolated using Image-J/FIJI thresholding, binary, and outline tools. ROIs were subsequently used to measure thickness, signal intensity, surface area, and perimeter length.

Confocal microscopy was carried out using a Nikon FN/C2 upright confocal microscope with a Coherent OBIS and Sapphire lasers (Nikon Instruments Inc., Melville, NY, United States; Coherent, Inc., Santa Clara, CA, United States). Corneal calcification was measured quantitatively after euthanasia using confocal microscopy imaging of corneal autofluorescence (Nikon, Melville NY). A total of 62 images (left and right eyes from 31 mice: 17 DBA/2J, 2 at 6 months, 5 at 9 months and 7 at 12 months of age, and 14 DBA/2J-*Gpnmb*^+^/SjJ control mice, 4 at 6 months, 5 at 9 months and 8 at 12 months of age) were obtained under identical conditions and were analyzed with Image-J/FIJI (National Institute of Health, United States). Each image was acquired as a Z-stack from each eye of each animal used for calcification measurement. Optical sectioning along the Z-axis was used to determine the thickness of calcified areas and maximum intensity projection images were generated to measure average signal intensity (a correlate of calcium crystal density), surface area, and perimeter length of calcified segments (Figure 2).

### Statistical Methods

All analyses were performed with GraphPad Prism Versions 8 and 9 (GraphPad Software, San Diego, CA), Microsoft Excel (Microsoft Corporation, Redmond, WA) and IBM SPSS Statistics Version 25 (IBM Corp., Armonk, NY). Pearson correlation coefficients were measured using bivariate correlations and strength of association was determined as follows: no correlation for 0.0 ≤ *r* < 0.2, weak correlation for 0.2 ≤ *r* < 0.4, moderate correlation for 0.4 ≤ *r* < 0.6, and strong correlation for 0.6 ≤ *r* ≤ 1.0. Sample means, mean differences, and 95% confidence intervals were obtained using independent *t*-tests, dependent *t*-tests, mixed-effects analyses and analyses of variance (ANOVAs). Tukey’s tests and Bonferroni corrections were used to account for multiple comparisons.

## Results

### Iris Pigment Dispersion—Comparisons Among Strains and Age Groups

At 6 months of age, 6.7% (4/60) of DBA/2J eyes demonstrated evidence of IPD. At 9 months of age, 77.5% (31/40) of DBA/2J eyes demonstrated evidence of IPD and at 12 months of age, 100% (18/18) of DBA/2J eyes showed evidence of IPD. No DBA/2J-*Gpnmb*^+^/SjJ mice developed IPD.

### Intraocular Pressure—Comparisons Among Strains and Age Groups

**FIGURE 3:**
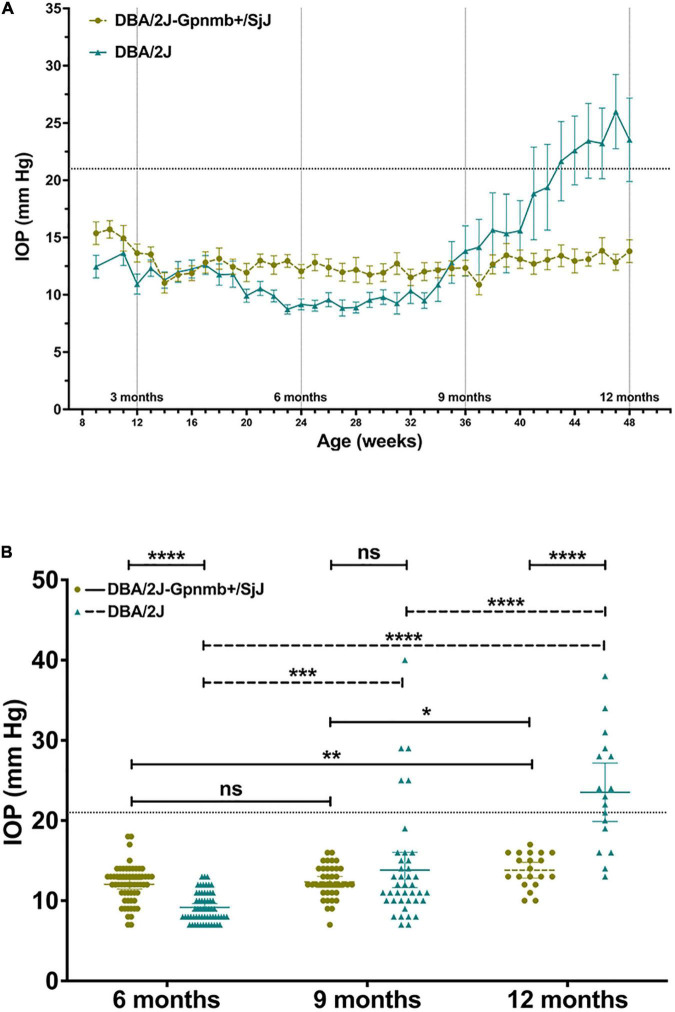
**(A)** Line graph illustrating mean intraocular pressure of DBA/2J and DBA/2J-*Gpnmb*^+^/SjJ mice by age. Mean intraocular pressure became consistently greater in DBA/2J mice than DBA/2J-*Gpnmb*^+^/SjJ at 41 weeks. **(B)** Column graph demonstrating intraocular pressure between groups and age. Line at 21 mm Hg indicates demarcation between normal tension and ocular hypertension. Mean intraocular pressure of DBA/2J and DBA/2J-*Gpnmb*^+^/SjJ mice increased at 12 months from 6 months. Mean intraocular pressure also increased at 9 months from 6 months in the DBA/2J group. *P < 0.05, **P < 0.01, ***P < 0.001, ****P < 0.0001. Error bars represent 95% confidence intervals. Sample sizes for A varied at each time point due to the availability of mice to be measured and ranged from 17 to 60 eyes for each point. Sample sizes for B were, from left to right (60, 60, 38, 40, 20, 17).

Mean IOP of DBA/2J-*Gpnmb*^+^/SjJ mice increased (*P* = 0.005; 95% CI, 0.480–3.02 mm Hg) at 12 months (13.8 ± 0.47 mm Hg) from 6 months (12.05 ± 0.30 mm Hg). Mean IOP increased at both 9 months (13.53 ± 0.88 mm Hg) and 12 months (23.53 ± 1.72 mm Hg) when compared with IOP measured at 6 months (9.3 ± 0.24 mm Hg; *P* < 0.001; 95% CI 2.22–6.23 and 11.57–16.89 mm Hg, respectively) in the DBA/2J group. At 41 weeks, IOP became consistently higher in DBA/2J mice (mean difference 6.15 ± 1.98 mm Hg; *P* = 0.003; Figure 3A) and at 12 months, mean IOP was significantly greater (*P* < 0.001; 95% CI, 5.99–13.47 mm Hg; Figure 3B) in DBA/2J mice (23.53 ± 1.72 mm Hg) when compared with DBA/2J-*Gpnmb*^+^/SjJ mice (13.8 ± 0.47 mm Hg; Figure 3B). While no DBA/2J-*Gpnmb*^+^/SjJ mice developed ocular hypertension, IOPs over 21 mm Hg were seen in DBA/2J mice for 5 out of 40 eyes (from 3 animals) at 9 months and in 11 out of 20 eyes (from 8 animals) at 12 months (Figure 3B).

**FIGURE 4:**
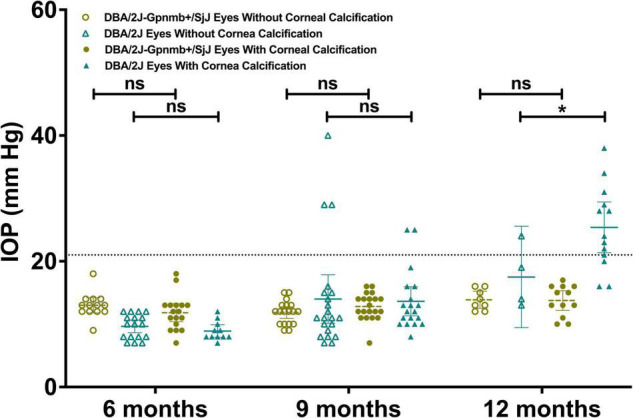
Column graph illustrating IOP in DBA/2J mice and DBA/2J-*Gpnmb*^+^/SjJ mice stratified by age and presence of corneal calcification. Line at 21 mm Hg indicates demarcation between normal tension and ocular hypertension. Mean intraocular pressure at 12 months was greater in calcified DBA/2J eyes when compared to non-calcified DBA/2J eyes. No difference in mean IOP was observed between DBA/2J-*Gpnmb*^+^/SjJ mice with or without corneal calcification. *P < 0.05. Error bars represent 95% confidence intervals. Sample sizes were, from left to right [(13, 17, 17, 11) (18, 21, 20, 19) (8, 4, 12, 13)].

Mean IOP at 12 months was greater (*P* = 0.048; 95% CI, 0.010–16.67 mm Hg) in DBA/2J eyes with corneal calcification (25.38 ± 1.85 mm Hg) when compared to DBA/2J eyes without corneal calcification (17.5 ± 2.53 mm Hg; Figure 4). No difference in mean IOP was observed between DBA/2J-*Gpnmb*^+^/SjJ mice with or without corneal calcification (Figure 4).

### Qualitative Assessment of Corneal Calcification—Comparisons Among Strains and Age Groups

**FIGURE 5:**
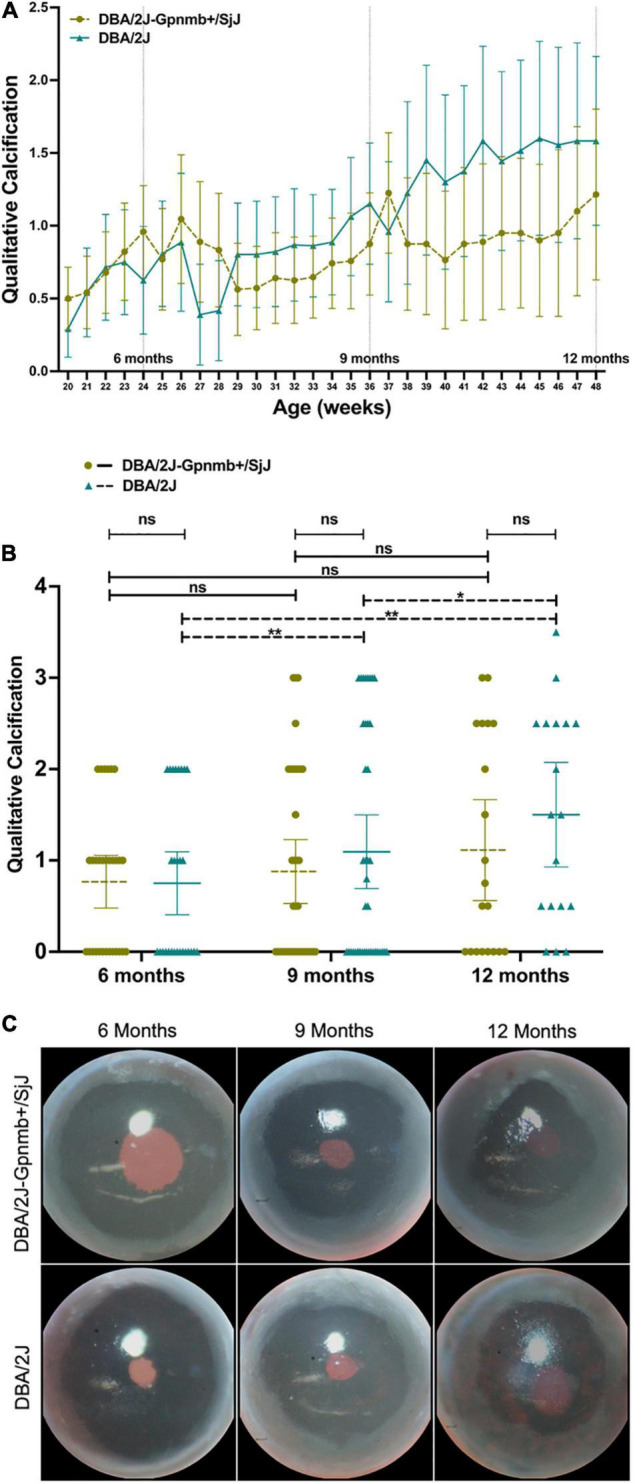
**(A)** Line graph, **(B)** column graph and **(C)** photographs illustrating the degree of qualitative corneal calcification in both DBA/2J and DBA/2J-*Gpnmb*^+^/SjJ mice euthanized at 12 months. The degree of qualitative calcification increased with age in both DBA/2J and DBA/2J-*Gpnmb*^+^/SjJ mice. No differences in qualitative calcification were observed between groups. *P < 0.05, **P < 0.01. Error bars represent 95% confidence intervals. Sample sizes for A varied at each time point due to the availability of mice to be measured and ranged from 18 to 40 eyes for each point. Sample sizes for B were, from left to right [(30, 28),(37, 40),(20, 18)].

Corneal calcification was found in 46.4% of DBA/2J eyes and 56.7% of DBA/2J-*Gpnmb*^+^/SjJ eyes at 6 months (*P* = 0.32), 52.5 and 52.6% at 9 months (*P* = 0.99) and 83.3 and 60.0% at 12 months (*P* = 0.11). Mixed-effects analysis demonstrated an increase in qualitative calcification with age in both DBA/2J (*P* = 0.049) and DBA/2J-*Gpnmb*^+^/SjJ mice (*P* = 0.04). While two-way ANOVA examining the effects of group and age on qualitative calcification revealed an interaction between groups (*P* = 0.03), multiple comparisons analyses revealed no individual differences between groups (Figures 5A–C).

### Objective Measures of Calcification—Comparisons Among Strains and Age Groups

**FIGURE 6:**
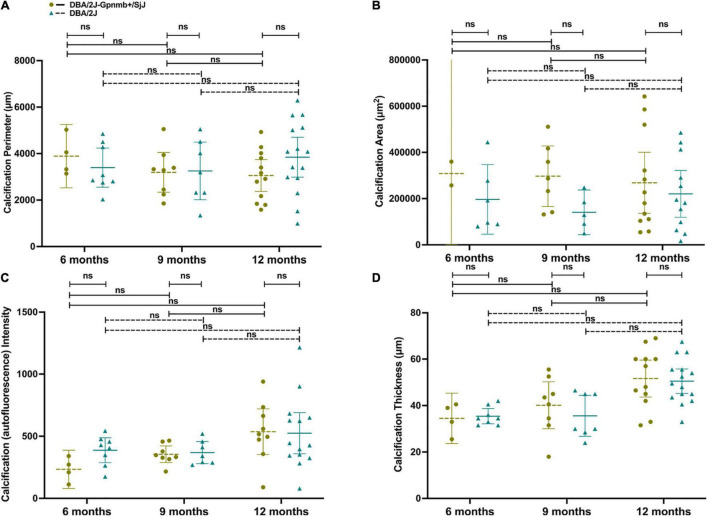
No differences in calcification **(A)** perimeter length, **(B)** area, **(C)** autofluorescence intensity, or **(D)** thickness were observed between groups or ages in eyes with cornea calcification. Error bars represent 95% confidence intervals. Sample sizes varied at each time point due to the number of mice with calcification. Sample sizes were from left to right A[(4, 8),(8, 7),(12, 15)], B[(2, 6),(7, 5),(12, 12)], C[(4, 8),(8,7),(9, 14)], D[(4, 8),(8, 7),(12, 15)].

Using objective measures of corneal calcification, we determined an increase in the number of eyes affected with age (Figure 6): calcification was found in 40% of DBA/2J eyes and 20% of DBA/2J-*Gpnmb*^+^/SjJ eyes at 6 months (*P* = 0.17), 35 and 47% at 9 months (*P* = 0.46), and 83 and 60% at 12 months (*P* = 0.11).

When comparing 9 and 12 month-old mice with corneal calcification, DBA/2J mice did not demonstrate an increase in calcification perimeter length (Figure 6A; *P* = 0.61), area (Figure 6B; *P* = 0.67), autofluorescence intensity (Figure 6C; *P* = 0.66) or thickness (Figure 6D; *P* = 0.07). Furthermore, DBA/2J-*Gpnmb*^+^/SjJ mice did not demonstrate changes in any of the aforementioned parameters between 9 and 12 months of age (*P* = 0.98, 0.98, 0.23, and 0.20, respectively; Figures 6A–D). Moreover, DBA/2J mice did not exhibit significantly greater mean calcification perimeter length at 12 months (Figure 6A; *P* = 0.35) or calcification intensity at 6 months (Figure 6C; *P* = 0.65) when compared to DBA/2J-*Gpnmb*^+^/SjJ mice of the same age. In sum, no differences in objective measures of calcification were observed between groups or ages (Figures 6A–D).

### Intraocular Pressure and Calcification—Comparisons Among Strains and Age Groups

**FIGURE 7:**
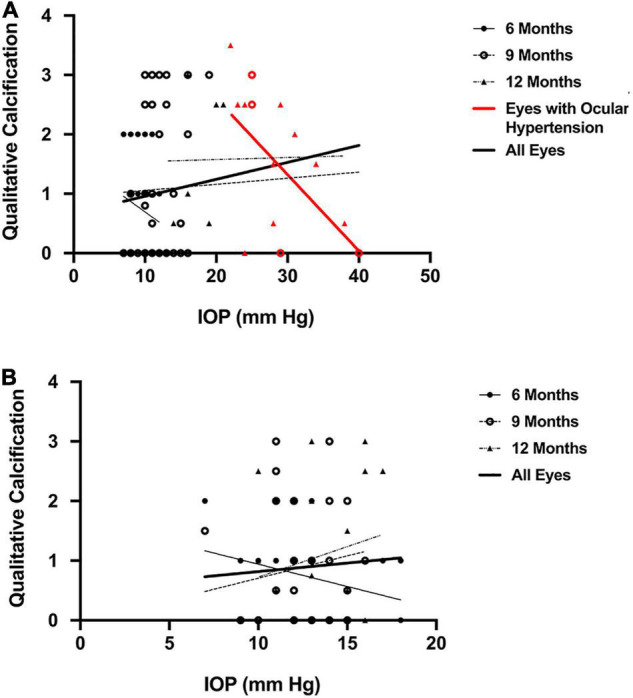
Scatterplot demonstrating **(A)** no correlation between qualitative calcification and intraocular pressure (r = 0.19) in all eyes of DBA/2J mice and **(B)** of DBA/2J-*Gpnmb*^+^/SjJ mice (*r* = 0.07). Qualitative calcification and intraocular pressure (r = –0.55, *P* = 0.03) were negatively correlated in eyes with ocular hypertension (highlighted in red). Sample sizes for each group in A were (6 mo = 28; 9 mo = 66, 12 mo = 18, Hypertensive = 15, All eyes = 86). Sample sizes for each group in B were (6 mo = 30; 9 mo = 38, 12 mo = 20, All eyes = 87).

IOP demonstrated no correlation with qualitative calcification (*r* = 0.19, *P* = 0.08; Figure 7A) when all eyes of DBA/2J mice were included. These parameters were also not correlated in DBA/2J-*Gpnmb*^+^/SjJ mice (*r* = 0.07, *P* = 0.54; Figure 7B) and for the three ages investigated in either strain (DBA/2J mice at 6, 9 and 12 months, *r* = –0.18, *P* = 0.37, *r* = 0.06, *P* = 0.73, *r* = 0.02, *P* = 0.94, respectively; DBA/2J-*Gpnmb*^+^/SjJ mice at 6, 9 and 12 months, *r* = 0.24, *P* = 0.20, *r* = 0.15, *P* = 0.38, *r* = 0.18, *P* = 0.44, respectively; Figure 7). For eyes with ocular hypertension (red symbols in Figure 7), which were only found in DBA/2J mice, IOP was negatively correlated with qualitatively assessed calcification (*r* = –0.55, *P* = 0.03; Figure 7A).

**FIGURE 8:**
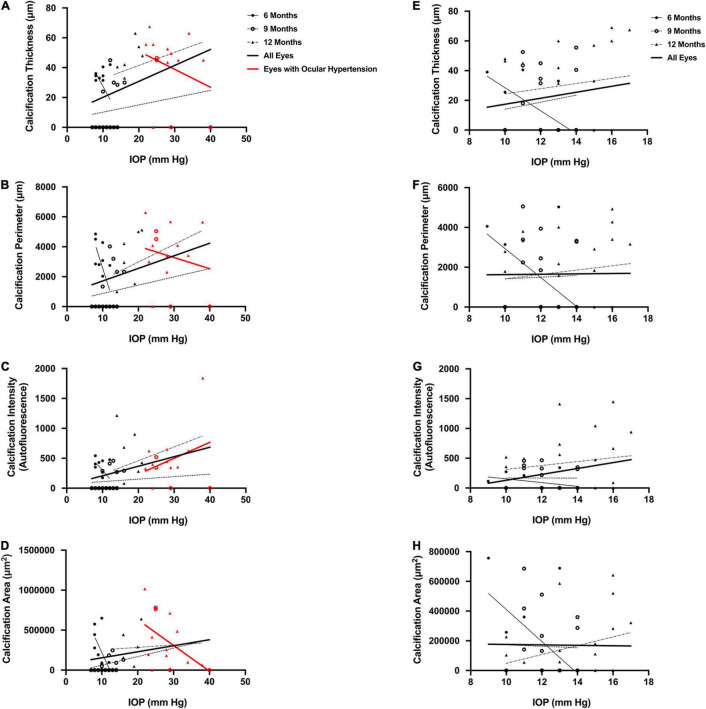
Scatterplots for DBA/2J mice demonstrating weak to moderate correlations between **(A)** intraocular pressure (IOP) and calcification thickness among all eyes (*r* = 0.42, *P* = 0.004), **(B)** IOP and calcification perimeter length among all eyes (*r* = 0.36, *P* = 0.01), and **(C)** IOP and calcification intensity among all eyes (*r* = 0.39, *P* = 0.007), but not **(D)** IOP and calcification area among all eyes (*r* = 0.25, *P* = 0.09). Scatterplots for *Gpnmb*^+^/SjJ mice demonstrate no relationship between **(E)** IOP and calcification thickness among all eyes (*r* = 0.16, *P* = 0.29), **(F)** IOP and calcification perimeter length among all eyes (*r* = 0.01, *P* = 0.95), **(G)** IOP and calcification intensity among all eyes (*r* = 0.26, *P* = 0.08), and **(H)** IOP and calcification area among all eyes (*r* = –0.01, *P* = 0.93). There were no significant positive correlations when mice in either strain were examined at 6, 9, and 12 months of age and there were no relationships between IOP and quantitative measures of calcification in mice with ocular hypertension. Error bars represent 95% confidence intervals. Sample sizes varied at each time point due to the number of mice with calcification. Sample sizes as in Figure 6.

For quantitative measures of corneal calcification, DBA/2J mice demonstrated weak to moderate correlations between IOP and calcification thickness (*r* = 0.42, *P* = 0.004; Figure 8A), IOP and calcification perimeter length (*r* = 0.36, *P* = 0.01; Figure 8B) and IOP and calcification intensity (*r* = 0.39, *P* = 0.007; Figure 8C) but not calcification area (*r* = 0.25, *P* = 0.09; Figure 8D). These parameters were not significantly positively correlated in DBA/2J-*Gpnmb*^+^/SjJ mice (*r* = 0.16, *P* = 0.29, *r* = 0.01, *P* = 0.95, *r* = 0.26, *P* = 0.08, *r* = –0.01, *P* = 0.93, respectively; Figures 8E–H) and for the three ages investigated in either strain (Figures 8A–H and Table 2).

**TABLE 2:** Correlations between intraocular pressure and quantitative measures of corneal calcification in DBA/2J and DBA/2J-*Gpnmb*^+^/SjJ mice.

Measure of corneal calcification	Pearson correlation coefficient (correlation with IOP)	*P-*value
**All DBA2J eyes (*n* = 48)**		
Thickness	0.42	0.004
Perimeter length	0.36	0.01
Intensity	0.39	0.007
Area	0.25	0.09
**DBA/2J eyes at 6 months (*n* = 10)**		
Thickness	–0.45	0.19
Perimeter length	–0.69	0.03
Intensity	–0.54	0.11
Area	–0.64	0.048
**DBA/2J eyes at 9 months (*n* = 20)**		
Thickness	0.23	0.33
Perimeter length	0.27	0.25
Intensity	0.19	0.42
Area	0.39	0.09
**DBA/2J eyes at 12 months (*n* = 18)**		
Thickness	0.33	0.20
Perimeter length	0.02	0.94
Intensity	0.36	0.16
Area	0.07	0.78
**Eyes with ocular hypertension (*n* = 14)**		
Thickness	–0.29	0.32
Perimeter length	–0.19	0.51
Intensity	0.33	0.26
Area	0.52	0.057
**All DBA/2J-*Gpnmb*^+^/SjJ eyes (*n* = 47)**		
Thickness	0.16	0.29
Perimeter length	0.01	0.95
Intensity	0.26	0.08
Area	–0.01	0.93
**DBA/2J-*Gpnmb*^+^/SjJ eyes at 6 months (*n* = 10)**		
Thickness	–0.65	0.04
Perimeter length	–0.54	0.11
Intensity	–0.36	0.31
Area	–0.54	0.10
**DBA/2J-*Gpnmb*^+^/SjJ eyes at 9 months (*n* = 17)**		
Thickness	0.13	0.61
Perimeter length	0.03	0.91
Intensity	–0.002	0.99
Area	–0.03	0.92
**DBA/2J-*Gpnmb*^+^/SjJ eyes at 12 months (*n* = 20)**		
Thickness	0.13	0.58
Perimeter length	0.13	0.58
Intensity	0.14	0.54
Area	0.30	0.20

*IOP, intraocular pressure.*

For eyes with ocular hypertension (red symbols in Figure 8), which were only found in DBA/2J mice, IOP was not correlated with quantitative measures of calcification (calcification thickness, *r* = –0.29, *P* = 0.32; Figure 8A; calcification perimeter length, *r* = –0.19, *P* = 0.51; Figure 8B; calcification intensity, *r* = 0.33, *P* = 0.26; Figure 8C; calcification area, *r* = 0.52, *P* = 0.057; Figure 8D).

### Intraocular Pressure and Iris Pigment Dispersion—Comparisons Among Strains and Age Groups

**FIGURE 9:**
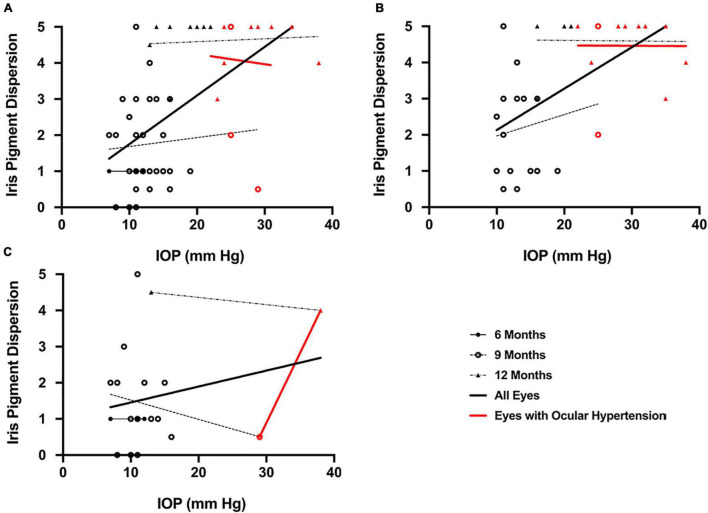
Scatterplots demonstrating a moderate correlation between **(A)** intraocular pressure (IOP) and qualitative iris pigment dispersion (IPD) in DBA/2J mice (*r* = 0.57, *P* < 0.001) which **(B)** increased when only mice with calcification were included in the analysis (*r* = 0.60, *P* < 0.001) and **(C)** was not detected when mice without calcification were excluded from analysis (*r* = 0.22). There were no significant correlations between IOP and qualitative IPD in mice with ocular hypertension. Error bars represent 95% confidence intervals. Sample sizes varied at each time point due to the number of mice with calcification. Sample sizes as in Figure 6.

IOP and the degree of IPD development were moderately correlated in DBA/2J mice (*r* = 0.57, *P* < 0.001; Figure 9A). This correlation was even stronger when only mice with corneal calcification were analyzed (*r* = 0.60, *P* < 0.001; Figure 9B) and was absent in the subset of mice that did not exhibit corneal calcification (*r* = 0.22, *P* = 0.33; Figure 9C). For the three ages investigated in DBA/2J mice, the correlation between IOP and the degree of IPD development did not increase with age (9 months: *r* = 0.09, *P* = 0.60; 12 months: *r* = 0.08, *P* = 0.76, respectively; Figure 9). For eyes with ocular hypertension (red symbols in Figure 9), which were only found in DBA/2J mice, IOP was not correlated with qualitatively assessed IPD development (*r* = –0.05, *P* = 0.91; Figure 9). IPD was not observed in DBA/2J-*Gpnmb*^+^/SjJ mice.

### Iris Pigment Dispersion and Corneal Calcification—Comparisons Among Age Groups

**FIGURE 10:**
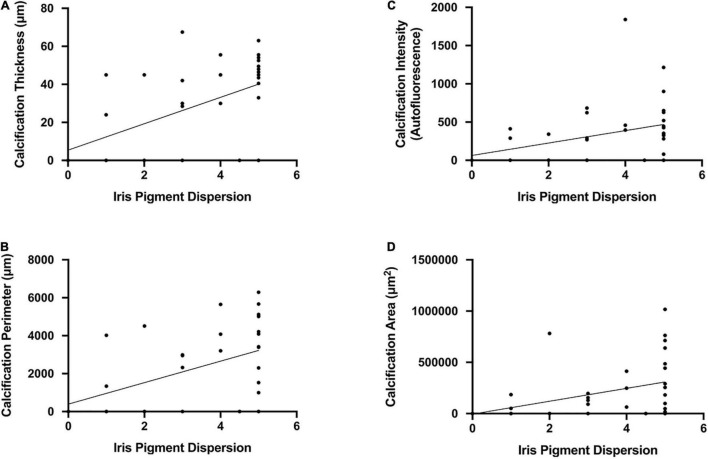
Scatterplots demonstrating weak to moderate correlations between iris pigment dispersion and **(A)** calcification thickness (*r* = 0.48, *P* = 0.004), **(B)** calcification perimeter (*r* = 0.47, *P* = 0.008), **(C)** calcification intensity (*r* = 0.34, *P* = 0.048), and **(D)** calcification area (*r* = 0.38, *P* = 0.03). Error bars represent 95% confidence intervals. Sample sizes varied at each time point due to the number of mice with calcification. Sample sizes as in Figure 6.

At time of euthanasia, DBA/2J mice demonstrated weak to moderate correlations between IPD and calcification thickness (*r* = 0.48, *P* = 0.004; Figure 10A), calcification perimeter (*r* = 0.47, *P* = 0.008; Figure 10B), calcification intensity (*r* = 0.34, *P* = 0.048; Figure 10C), calcification area (*r* = 0.38, *P* = 0.03; Figure 10D), and qualitative calcification (*r* = 0.35, *P* = 0.04). No correlations were observed in DBA2/J mice when IPD and corneal calcification were examined by individual age cohorts (6, 9, and 12 months).

## Discussion

In the present study, we used DBA/2J-*Gpnmb*^+^/SjJ mice as a control for DBA/2J mice to characterize anterior segment abnormalities in the context of the development of elevated IOP and ocular hypertension. The genotype of DBA/2J-*Gpnmb*^+^/SjJ mice is identical to the DBA/2J genotype with the exception of a functional allele that prevents the development of IPD and thus of an elevated IOP (Howell et al., 2007). Therefore, it is assumed that DBA/2J-*Gpnmb*^+^/SjJ mice serve as an appropriate control strain to evaluate the role of elevated IOP in the development of visual dysfunction characteristic of the DBA/2J mouse. However, the use of a rebound tonometer in the presence of corneal calcification on the DBA/2J mouse prompted the question of whether the increase in IOP was a result of an artificial elevation. To evaluate whether increased IOP in DBA/2J was due to IPD or corneal calcification the DBA/2J-*Gpnmb*^+^/SjJ strain would need to have reduced or absent IPD, reduced or absent elevation in IOP, and similar levels of corneal calcification.

### Only DBA/2J Mice, but Not DBA/2J-*Gpnmb*^+^/SjJ Mice, Exhibit IPD and Clinically Significant Increases in Intraocular Pressure

We confirmed that DBA/2J-*Gpnmb*^+^/SjJ mice do not develop IPD (Howell et al., 2007) by age 12 months, a time point when all DBA/2J mice in the present study showed evidence of IPD in both eyes. IPD is considered a principal contributor to the development of elevated IOP through the obstruction of aqueous outflow in DBA/2J mice (John et al., 1998; Libby et al., 2005; Scholz et al., 2008). We identified ocular hypertension in DBA/2J mice, where IOP increased on average by approximately 2.5 fold from age 6 to 12 months (Figure 3). IOP increased in a statistically significant manner from 6 to 9 months of age and then again more pronounced from 9 to 12 months in DBA/2J mice, with ocular hypertension developing and continuing after 43 weeks of age (Figure 3), a time-course pattern of IOP increase that is consistent with previous reports (Libby et al., 2005; Williams et al., 2013; Wang and Dong, 2016) and that is absent in DBA/2J-*Gpnmb*^+^/SjJ mice.

Previous studies have demonstrated the absence of an age-related IOP elevation in DBA/2J-*Gpnmb*^+^/SjJ mice (Howell et al., 2007). The present study confirmed this for the first 9 months of life, while we observed a statistically significant increase in IOP in the last quarter of the 12-month observation period. We observed a modest average increase in IOP of approximately 1 mm Hg in DBA/2J-*Gpnmb*^+^/SjJ mice by age 12 months. While this finding itself was statistically significant it appears biologically and clinically not significant as it does not lead to ocular hypertension (Figures 3, 4). The lack of significantly elevated IOP, and the lack of IPD supports the use of the DBA/2J-*Gpnmb*^+^/SjJ strain as a suitable control animal for the DBA/2J strain up to 12 months of age.

### The Degree of Corneal Calcification Does Not Differ Between DBA/2J and DBA/2J-*Gpnmb*^+^/SjJ Mice

At the same time, we measured a moderate but clinically relevant generalized increase in qualitative corneal calcification with age in both DBA/2J and DBA/2J-*Gpnmb*^+^/SjJ mice (Figure 5), where at age 12 months the degree of corneal calcification was not statistically significantly different between the two strains when assessed qualitatively (Figure 5B) or quantitatively (Figures 6A–D).

Elevated IOP characteristic of the DBA/2J mouse strain has previously been attributed to blockage of the aqueous humor pathway due to IPD, peripheral synechiae, and iris atrophy (John et al., 1998; Chang et al., 1999; Anderson et al., 2002; Libby et al., 2005; Howell et al., 2007; Scholz et al., 2008). While corneal thickness has been associated with elevated IOP in DBA/2J mice (Inman et al., 2006; Chou et al., 2011), corneal calcification, although previously described (John et al., 1998; Bricker-Anthony and Rex, 2015; Turner et al., 2017), has not been studied in the context of elevated IOP. Furthermore, the presence and extent of corneal calcification has not been evaluated in DBA/2J-*Gpnmb*^+^/SjJ mice.

Not surprisingly, nearly identical incidences of corneal calcification were seen in both DBA/2J and DBA/2J-*Gpnmb*^+^/SjJ mice (Figure 5). These data indicate that the genetic alterations introduced into the DBA/2J-*Gpnmb*^+^/SjJ strain do not affect corneal calcification as expected. The lack of significant differences in corneal calcification supports the use of the DBA/2J-*Gpnmb*^+^/SjJ strain as a suitable control animal for the DBA/2J strain up to 12 months of age. DBA/2J mice demonstrated a statistically significant increase in the qualitative (Figure 5B) but not quantitative measures of corneal calcification (Figure 6) from 9 to 12 months while differences among the three ages of DBA/2J-*Gpnmb*^+^/SjJ mice were not statistically significant (Figures 5B, 6). However, we observed no statistically significant differences in qualitative and quantitative measures of corneal calcification when comparing DBA/2J to DBA/2J-*Gpnmb*^+^/SjJ mice (Figures 5B, 6). In sum, while not statistically significantly increasing over time, DBA/2J-*Gpnmb*^+^/SjJ mice nevertheless display corneal calcification at age 12 months that is not different from that in DBA/2J mice.

When the dependence of IOP on the degree of corneal calcification was analyzed in DBA/2J mice, a trend toward a correlation with increased IOP using qualitative measures was found, though it was not statistically significant (Figure 7). Quantitative measures of calcification were significantly correlated with IOP in DBA/2J mice (Figure 8). However, for the subset of eyes in DBA/2J mice that developed ocular hypertension, IOP was not positively correlated with measures of calcification (Figures 7, 8). DBA/2J-*Gpnmb*^+^/SjJ mice did not demonstrate significant increases in IOP with increases in qualitative (Figure 7) or quantitative measures of calcification (Figure 8) and at 12 months of age the degree of corneal calcification in DBA/2J-*Gpnmb*^+^/SjJ mice was statistically not different from that in DBA/2J mice (Figures 5, 6).

Taken together these findings indicate that while increasing levels of IPD as an anterior chamber abnormality are highly correlated with the development of increased IOP and ultimately ocular hypertension, the development of corneal calcification as an anterior chamber abnormality in both DBA/2J-*Gpnmb*^+^/SjJ and DBA/2J mice was independent of changes in IOP over time.

### Limitations and Future Studies

A limitation of the present study is the lack of an invasive cannulation IOP measurement to confirm the results of the rebound tonometer measurements. While it would have been insightful to identify whether the increase in IOP in the DBA/2J was influenced by the method of IOP determination, rebound tonometry is a widely accepted and validated method and ocular hypertension in the DBA/2J strain is well documented. Furthermore, the method of measurement does not affect our primary observation that corneal calcification does not cause elevated IOP readings. This is evident by the consistent, longitudinal, IOP measurements in both strains, consistently elevated IOP in the DBA/2J, a lack of ocular hypertension in the DBA/2J-*Gpnmb*^+^/SjJ strain combined with similar corneal calcification in both strains. As we only evaluated mice up to 12 months of age, further investigation should seek to evaluate the role of corneal calcification on IOP beyond this age.

In addition, given the small sample size of the present study and the variability in disease progression seen for the DBA/2J strain, statistically significant results from the present study suggest that future, larger sized studies consider the potential impact of corneal calcification in both the DBA/2J and the DBA/2J-*Gpnmb*^+^/SjJ strain on relevant preclinical outcome measures other than IOP.

## Summary

Previous reports indicated that DBA/2J mice exhibit falsely elevated IOP when measured non-invasively (Turner et al., 2017). One possible explanation for this artifact was the presence of calcium deposits on the cornea disrupting rebound tonometry readings. In the present study we have shown in the DBA/2J-*Gpnmb*^+^/SjJ strain, a strain derived from the DBA/2J strain to lack IPD, that even in the presence of significant corneal calcification IOP measurement stays consistent and is not artificially elevated. While there was a positive correlation between calcification and IOP in the 12-month-old DBA/2J animals, similar levels of calcification in the 12-month-old sub-groups combined with a lack of elevated IOP in the DBA/2J-*Gpnmb*^+^/SjJ control strain does not support a causative relationship between these factors. These data strongly support the argument that corneal calcification does not cause false readings of IOP measured non-invasively and support the use of the DBA/2J-*Gpnmb*^+^/SjJ animal as an ideal control animal to be paired with the DBA/2J mouse to investigate diseases of the eye related to elevated intraocular pressure.

The DBA/2J mouse strain has been widely used as a model for ocular hypertension and glaucoma due to the presence of progressively worsening anterior chamber abnormalities and IOP increasing with age (Sheldon et al., 1995; Inman et al., 2006; Schlamp et al., 2006; Burroughs et al., 2011; Bosco et al., 2018; Mathieu et al., 2018; Buchanan et al., 2019; Jassim et al., 2019). In this study, we used strain-matched DBA/2J-*Gpnmb*^+^/SjJ mice to determine the relative contributions of corneal calcification and IPD to the apparent elevation of IOP characteristic of the DBA/2J mouse strain.

## Data Availability Statement

The original contributions presented in the study are included in the article. Further inquiries can be directed to the corresponding author.

## Ethics Statement

The animal study was reviewed and approved by the University of Missouri—Kansas City, Institutional Animal Care and Use Committee.

## Author Contributions

PK conceived and designed the experiments. LR, RD, and MM performed the experiments. LR and PK wrote the manuscript. All authors analyzed the data, edited, and reviewed the manuscript.

## Conflict of Interest

The authors declare that the research was conducted in the absence of any commercial or financial relationships that could be construed as a potential conflict of interest.

## Publisher’s Note

All claims expressed in this article are solely those of the authors and do not necessarily represent those of their affiliated organizations, or those of the publisher, the editors and the reviewers. Any product that may be evaluated in this article, or claim that may be made by its manufacturer, is not guaranteed or endorsed by the publisher.

## Abbreviations

ANOVA: analysis of variance
IOP: intraocular pressure
IPD: iris pigment dispersion
ISA: iris stromal atrophy.

## References

[B1] Anderson M. G. Smith R. S. Hawes N. L. Zabaleta A. Chang B. Wiggs J. L. (2002). Mutations in genes encoding melanosomal proteins cause pigmentary glaucoma in DBA/2J mice. *Nat. Genet.* 30 81–85. 10.1038/ng794 11743578

[B2] Bosco A. Anderson S. R. Breen K. T. Romero C. O. Steele M. R. Chiodo V. A. (2018). Complement C3-Targeted gene therapy restricts onset and progression of neurodegeneration in chronic mouse glaucoma. *Mol. Ther.* 26 2379–2396. 10.1016/j.ymthe.2018.08.017 30217731 PMC6171099

[B3] Brandt J. D. (2001). The influence of corneal thickness on the diagnosis and management of glaucoma. *J. Glaucoma* 10(5 Suppl. 1), S65–S67. 10.1097/00061198-200110001-200110023 11890281

[B4] Bricker-Anthony C. Rex T. S. (2015). Neurodegeneration and vision loss after mild blunt trauma in the C57Bl/6 and DBA/2J mouse. *PLoS One* 10:e0131921. 10.1371/journal.pone.0131921 26148200 PMC4493046

[B5] Buchanan R. A. Foley K. E. Pepper K. W. Reagan A. M. Keezer K. J. Hewes A. A. (2019). Meox2 haploinsufficiency accelerates axonal degeneration in DBA/2J glaucoma. *Invest. Ophthalmol. Vis. Sci.* 60 3283–3296. 10.1167/iovs.18-26126 31369031 PMC6676925

[B6] Burroughs S. L. Kaja S. Koulen P. (2011). Quantification of deficits in spatial visual function of mouse models for glaucoma. *Invest. Ophthalmol. Vis. Sci.* 52 3654–3659. 10.1167/iovs.10-7106 21330670 PMC3109046

[B7] Chang B. Smith R. S. Hawes N. L. Anderson M. G. Zabaleta A. Savinova O. (1999). Interacting loci cause severe iris atrophy and glaucoma in DBA/2J mice. *Nat. Genet.* 21 405–409. 10.1038/7741 10192392

[B8] Chou T. H. Kocaoglu O. P. Borja D. Ruggeri M. Uhlhorn S. R. Manns F. (2011). Postnatal elongation of eye size in DBA/2J mice compared with C57BL/6J mice: in vivo analysis with whole-eye OCT. *Invest. Ophthalmol. Vis. Sci.* 52 3604–3612. 10.1167/iovs.10-6340 21372015 PMC3109044

[B9] Grillo S. L. Koulen P. (2015). Psychophysical testing in rodent models of glaucomatous optic neuropathy. *Exp. Eye Res.* 141 154–163. 10.1016/j.exer.2015.06.025 26144667 PMC4628867

[B10] Grillo S. L. Keereetaweep J. Grillo M. A. Chapman K. D. Koulen P. (2013). N-Palmitoylethanolamine depot injection increased its tissue levels and those of other acylethanolamide lipids. *Drug Des. Dev. Ther.* 7 747–752. 10.2147/DDDT.S48324 23976843 PMC3746786

[B11] Howell G. R. Libby R. T. Marchant J. K. Wilson L. A. Cosma I. M. Smith R. S. (2007). Absence of glaucoma in DBA/2J mice homozygous for wild-type versions of Gpnmb and Tyrp1. *BMC Genet.* 8:45. 10.1186/1471-2156-8-45 17608931 PMC1937007

[B12] Inman D. M. Sappington R. M. Horner P. J. Calkins D. J. (2006). Quantitative correlation of optic nerve pathology with ocular pressure and corneal thickness in the DBA/2 mouse model of glaucoma. *Invest. Ophthalmol. Vis. Sci.* 47 986–996. 10.1167/iovs.05-0925 16505033

[B13] Jackson H. M. Onos K. D. Pepper K. W. Graham L. C. Akeson E. C. Byers C. (2015). DBA/2J genetic background exacerbates spontaneous lethal seizures but lessens amyloid deposition in a mouse model of Alzheimer’s disease. *PLoS One* 10:e0125897. 10.1371/journal.pone.0125897 25933409 PMC4416920

[B14] Jassim A. H. Coughlin L. Harun-Or-Rashid M. Kang P. T. Chen Y. R. Inman D. M. (2019). Higher reliance on glycolysis limits glycolytic responsiveness in degenerating glaucomatous optic nerve. *Mol. Neurobiol.* 56 7097–7112. 10.1007/s12035-019-1576-1574 30980229 PMC6728180

[B15] John S. W. Smith R. S. Savinova O. V. Hawes N. L. Chang B. Turnbull D. (1998). Essential iris atrophy, pigment dispersion, and glaucoma in DBA/2J mice. *Invest. Ophthalmol. Vis. Sci.* 39 951–962. 9579474

[B16] Kaja S. Naumchuk Y. Grillo S. L. Borden P. K. Koulen P. (2014). Differential up-regulation of Vesl-1/Homer 1 protein isoforms associated with decline in visual performance in a preclinical glaucoma model. *Vision Res.* 94 16–23. 10.1016/j.visres.2013.10.018 24219919 PMC3890355

[B17] Libby R. T. Anderson M. G. Pang I. H. Robinson Z. H. Savinova O. V. Cosma I. M. (2005). Inherited glaucoma in DBA/2J mice: pertinent disease features for studying the neurodegeneration. *Vis. Neurosci.* 22 637–648. 10.1017/S0952523805225130 16332275

[B18] Mathieu E. Gupta N. Paczka-Giorgi L. A. Zhou X. Ahari A. Lani R. (2018). Reduced cerebrospinal fluid inflow to the optic nerve in glaucoma. *Invest. Ophthalmol. Vis. Sci.* 59 5876–5884. 10.1167/iovs.18-24521 30543343

[B19] Montgomery C. L. Keereetaweep J. Johnson H. M. Grillo S. L. Chapman K. D. Koulen P. (2016). Changes in retinal N-Acylethanolamines and their oxylipin derivatives during the development of visual impairment in a mouse model for glaucoma. *Lipids* 51 857–866. 10.1007/s11745-016-4161-x 27221132 PMC4911801

[B20] Pease M. E. Cone F. E. Gelman S. Son J. L. Quigley H. A. (2011). Calibration of the TonoLab tonometer in mice with spontaneous or experimental glaucoma. *Invest. Ophthalmol. Vis. Sci.* 52 858–864. 10.1167/iovs.10-5556 20720229 PMC3053110

[B21] Rutsch F. Nitschke Y. Terkeltaub R. (2011). Genetics in arterial calcification: pieces of a puzzle and cogs in a wheel. *Circ. Res.* 109 578–592. 10.1161/CIRCRESAHA.111.247965 21852556 PMC3248761

[B22] Schlamp C. L. Li Y. Dietz J. A. Janssen K. T. Nickells R. W. (2006). Progressive ganglion cell loss and optic nerve degeneration in DBA/2J mice is variable and asymmetric. *BMC Neurosci.* 7:66. 10.1186/1471-2202-7-66 17018142 PMC1621073

[B23] Scholz M. Buder T. Seeber S. Adamek E. Becker C. M. Lutjen-Drecoll E. (2008). Dependency of intraocular pressure elevation and glaucomatous changes in DBA/2J and DBA/2J-Rj mice. *Invest. Ophthalmol. Vis. Sci.* 49 613–621. 10.1167/iovs.07-0745 18235006

[B24] Sheldon W. G. Warbritton A. R. Bucci T. J. Turturro A. (1995). Glaucoma in food-restricted and ad libitum-fed DBA/2NNia mice. *Lab Anim. Sci.* 45 508–518. 8569148

[B25] Shin J. B. Longo-Guess C. M. Gagnon L. H. Saylor K. W. Dumont R. A. Spinelli K. J. (2010). The R109H variant of fascin-2, a developmentally regulated actin crosslinker in hair-cell stereocilia, underlies early-onset hearing loss of DBA/2J mice. *J. Neurosci.* 30 9683–9694. 10.1523/JNEUROSCI.1541-10.2010 20660251 PMC2922854

[B26] Swaminathan S. Lu H. Williams R. W. Lu L. Jablonski M. M. (2013). Genetic modulation of the iris transillumination defect: a systems genetics analysis using the expanded family of BXD glaucoma strains. *Pigment Cell Melanoma Res.* 26 487–498. 10.1111/pcmr.12106 23582180 PMC3752936

[B27] Tham Y. C. Li X. Wong T. Y. Quigley H. A. Aung T. Cheng C. Y. (2014). Global prevalence of glaucoma and projections of glaucoma burden through 2040: a systematic review and meta-analysis. *Ophthalmology* 121 2081–2090. 10.1016/j.ophtha.2014.05.013 24974815

[B28] Turner A. J. Vander Wall R. Gupta V. Klistorner A. Graham S. L. (2017). DBA/2J mouse model for experimental glaucoma: pitfalls and problems. *Clin. Exp. Ophthalmol.* 45 911–922. 10.1111/ceo.12992 28516453

[B29] van den Broek F. A. Beynen A. C. (1998). The influence of dietary phosphorus and magnesium concentrations on the calcium content of heart and kidneys of DBA/2 and NMRI mice. *Lab. Anim.* 32 483–491. 10.1258/002367798780599758 9807763

[B30] Wang J. Dong Y. (2016). Characterization of intraocular pressure pattern and changes of retinal ganglion cells in DBA2J glaucoma mice. *Int. J. Ophthalmol.* 9 211–217. 10.18240/ijo.2016.02.05 26949637 PMC4761729

[B31] Williams P. A. Howell G. R. Barbay J. M. Braine C. E. Sousa G. L. John S. W. (2013). Retinal ganglion cell dendritic atrophy in DBA/2J glaucoma. *PLoS One* 8:e72282. 10.1371/journal.pone.0072282 23977271 PMC3747092

[B32] Wong A. A. Brown R. E. (2007). Age-related changes in visual acuity, learning and memory in C57BL/6J and DBA/2J mice. *Neurobiol. Aging* 28 1577–1593. 10.1016/j.neurobiolaging.2006.07.023 17010477

[B33] Yang X. L. van der Merwe Y. Sims J. Parra C. Ho L. C. Schuman J. S. (2018). Age-related changes in eye, brain and visuomotor behavior in the DBA/2J mouse model of chronic glaucoma. *Sci. Rep.* 8:4643. 10.1038/s41598-018-22850-22854 29545576 PMC5854610

